# miR-33b-3p Acts as a Tumor Suppressor by Targeting *DOCK4* in Prostate Cancer

**DOI:** 10.3389/fonc.2021.740452

**Published:** 2021-11-03

**Authors:** Yu Mei, Kai Li, Zhicheng Zhang, Mengmeng Li, Hong Yang, Hui Wang, Xuemei Huang, Xinyuan Li, Shuhua Shi, Huanjie Yang

**Affiliations:** ^1^ School of Life Science and Technology, Harbin Institute of Technology, Harbin, China; ^2^ Fourth Affiliated Hospital of Harbin Medical University, Harbin, China

**Keywords:** miR-33b-3p, DOCK4, metastasis, prostate cancer, proteasome inhibitor

## Abstract

Despite that androgen-deprivation therapy results in long-lasting responses, the disease inevitably progresses to metastatic castration-resistant prostate cancer. In this study, we identified miR-33b-3p as a tumor suppressor in prostate cancer. miR-33b-3p was significantly reduced in prostate cancer tissues, and the low expression of miR-33b-3p was correlated with poor overall survival of prostate cancer patients. Overexpression of miR-33b-3p inhibited both migration and invasion of highly metastatic prostate cancer cells whereas inhibition of miR-33b-3p promoted those processes in lowly metastatic cells. The *in vivo* results demonstrate that miR-33b-3p suppresses metastasis of tail vein inoculated prostate cancer cells to lung and lymph nodes in mice. *DOCK4* was validated as the direct target of miR-33b-3p. miR-33b-3p decreased the expression of *DOCK4* and restoration of *DOCK4* could rescue miR-33b-3p inhibition on cell migration and invasion. Moreover, downregulation of miR-33b-3p was induced by bortezomib, the clinically used proteasome inhibitor, and overexpression of miR-33b-3p enhanced the insufficient inhibition of bortezomib on migration and invasion as well as metastasis of prostate cancer cells. In summary, our findings demonstrate that miR-33b-3p suppresses metastasis by targeting *DOCK4* in prostate cancer. Our results suggest that enhancing miR-33b-3p expression may provide a promising therapeutic strategy for overcoming that proteasome inhibitor’s poor efficacy against metastatic prostate cancer.

## Introduction

Prostate cancer is the most common malignancies and the second leading cause of cancer-associated mortality in men (1). Despite that androgen-deprivation therapy results in long-lasting responses, the disease inevitably progresses to metastatic castration-resistant prostate cancer (mCRPC) that is associated with poor prognosis (2, 3). The proteasome is a validated target for cancer therapy. The first-in-class proteasome inhibitor bortezomib demonstrates great success against multiple myeloma (4). However, limited efficacy of bortezomib alone was observed in solid tumors including prostate cancer. As a combination regimen, bortezomib was found not able to improve prostate cancer patients’ response to docetaxel, a standard treatment for mCRPC (5, 6).

MicroRNAs (miRNAs) are a conserved class of small noncoding RNAs that have been recognized as key regulators of various cellular processes at post-transcriptional level (7, 8). Dysregulation of miRNAs has been demonstrated to broadly associate with tumor invasion and angiogenesis as well as drug resistance (7–11). Proteasome inhibitor leads to alteration in not only the proteins but also the miRNAs (12, 13). Our previous studies showed that miR-33b-3p was downregulated in prostate cancer in response to proteasome inhibition (14). It drew our attention that miR-33b-5p was predominantly upregulated in multiple myeloma post treatment with proteasome inhibitor (15, 16). Both arms of the miRNAs can be expressed as a mature form *in vivo* (17, 18). In some cases, they have similar function while in some cases; they work opposite (19). It has been shown that miR-33b-5p functions as tumor suppressor in prostate cancer (20). miR-33b-3p also functions as tumor suppressor in some type of cancers (21, 22). Thus, we speculated that miR-33b-3p might function as a tumor suppressor in prostate cancer, which downregulation in response to bortezomib might contribute to insufficient efficacy of targeting the proteasome in prostate cancer.

In the present study, we found that miR-33b-3p was the dominant mature form in prostate cancer. Elevated miR-33b-3p significantly reduced the metastasis of prostate cancer cells by targeting *DOCK4*. Moreover, downregulation of miR-33b-3p post bortezomib treatment was associated the insufficient inhibition on prostate cancer cells migration and invasion.

## Materials and Methods

### Cell Culture

Prostate and lung cancer cells were cultured in RPMI 1640 medium with 10% fetal bovine serum (FBS, Gibco-BRL, Grand Island, NY, USA) and breast cancer cells were cultured in DMEM medium with 10% FBS in a humid atmosphere containing 5% CO_2_ at 37°C.

### Primers and Oligos

Primers were synthesized by Comate Biosciences (Jilin, China). The mimic and inhibitor of miR-33b-3p were obtained from Ribio (Guangzhou, China). The primer and oligo sequences are listed in **Table 1**.

**Table 1:** Oligos used for PCR and vector construction.

Reverse Transcription primers	Sequences (5’-3’)
**miR-33b-3p**	*CTCAACTGGTGTCGTGGAGTCGGCAATTCAGTTGAGGG* GCTGCA
**miR-33b-5p**	CTCAACTGGTGTCGTGGAGTCGGCAATTCAGTTGAGGC AATGCA
**qRT-PCR primers**	**Sequences (5’-3’)**
**miR-33b-3p**	Forward: ACACTCCAGCTGGGCAGTGCCTCGGCAGTG
	Reverse: TGGTGTCGTGGAGTCG
**miR-33b-5p**	Forward: ACACTCCAGCTGGGGTGCATTGCTGTTG
	Reverse: TGGTGTCGTGGAGTCG
**U6**	Forward: CGCTTCGGCAGCACATATACTA
	Reverse: CGCTTCACGAATTTGCGTGTCA
**DOCK4**	Forward: GACCCACACACAGACTGCTTCA
	Reverse: GAGAGGGGGTGAAAGACTGC
**GAPDH**	Forward: TGCACCACCAAACTGCTTAGC
	Reverse: GGCATGGACTGTGGTCATGAG
**Plasmids construction Primers**	**Sequences (5’-3’)**
**pMIR-WT-3’ UTR**	Forward: GACTAGTTTTCTATGTACCTGCGATGC
	Reverse: CGACGCGTACGGTGTCAGGGTAGTAAGG
**pMIR-MUT-3’ UTR-1**	Forward: TTTAGTGTCGTCGCGTTAATAACTT
	Reverse: CGACGCGTACGGTGTCAGGGTAGTAAGG
**pMIR-MUT-3’ UTR-2**	Forward: TAAGTTATTAACGCGACGACACTAA
	Reverse: TAGTTGACCGCTTGAAGTCT
**HA-DOCK4-1**	Forward: AAACTCGAGATGTGGATACCTACGGAGCACGAG
	Reverse: GCATCAGAGGGACAAAAGAAAACC
**HA-DOCK4-2**	Forward: CGATGTTCATTGTAGACAGTAGTG
	Reverse: CCATTAGACGAGTTACAGTAGCA
**HA-DOCK4-3**	Forward: AGAAAAAGGTGTTAGAAAAGTATGG
	Reverse: AAAGCGGCCGCTTATAACTGAGAGACCTTGCGGGGC
**miRNA construction oligos**	**Sequences (5’-3’)**
**pLKO.1-miR-33b-3p**	Sense: CCGGCAGTGCCTCGGCAGTGCAGCCCCTCGAGGGGCT GCACTGCCGAGGCACTGTTTTTG
	Antisense: AATTCAAAAACAGTGCCTCGGCAGTGCAGCCCCTCGAG GGGCTGCACTGCCGAGGCACTG

### RT-qPCR

Total RNAs were isolated from culture cells or frozen prostate cancer tissues using Trizol (Invitrogen, Carlsbad, CA, USA) and reverse-transcribed (RT) into cDNA (500 ng total RNA) using ReverTra Ace (Toyobo, Kita-Ku, Osaka, Japan). Quantitative real-time PCR (RT-qPCR) was performed using SYBR Premix Ex Taq (Takara, Otsu, Shiga, Japan). The efficiency of primer for both arms of miR-33b (E) was determined from the slope of the curve obtained with the serially diluted standards (10^8^-10^5^ gene copies per qPCR reaction), as E = 10^(−1/slope)^ (23). The slopes of the calibration curves were calculated from the plot of the log base 10 of initial gene copy number *versus* corresponding threshold cycles (Ct values) and used to determine the number of gene copies in each sample. Microarray gene profiling datasets of GEO database was used to compare the expressions of both arms of miR-33b in normal prostate tissues and prostate cancer samples (http://www.ncbi.nlm.nih.gov/geo, GEO accession: GSE64318).

### Plasmid and Transfection

The wild type of 3’ UTR of *DOCK4* was amplified by PCR and cloned into the SpeI/MluI (Takara, Otsu, Shiga, Japan) sites of pMIR-REPORT vector (Promega, Madison, WI, USA). The mutated 3’ UTR of *DOCK4* was amplified using two-stepwise PCR and cloned into the same sites of pMIR-REPORT vector. The full length of *DOCK4* was amplified by overlapping PCR using three pairs of primers and cloned into the XhoI/NotI (Takara, Otsu, Shiga, Japan) sites of pIRM-3xHA (HA) vector (BCCM, Zwijnaarde, Gent, Belgium). The primer sequences are listed in **Table 1**. All these constructed plasmids were confirmed by sequencing. Transient transfection of miRNA or plasmids were mediated by Lipofectamine 3000 (Invitrogen, Carlsbad, CA, USA) reagent following the manufacturer’s protocol.

### Stable Cell Lines

pLKO.1-puro plasmid (Addgene, Watertown, MA, USA) was used for miR-33b-3p overexpression. Sense and antisense of miRNA oligos listed in **Table 1** were annealed and inserted into the Age I/EcoRI (Takara, Otsu, Shiga, Japan) digested pLKO.1-puro vector. Stable cell lines were established through **lentiviral** transduction. Briefly, lentiviral packaging mix (VSV-G plasmid and Gag-Pol plasmid) and constructed pLKO.1 plasmid were co-transfected into HEK293T cells. The supernatants containing lentiviruses were collected, filtered, and added into indicated cells. After 48 h of incubation with lentiviruses, the transduced cells were selected with puromycin (Santa Cruz, TX, USA). The knockdown efficiency or overexpression level was confirmed by RT-qPCR. Cells stably expressing firefly luciferase were confirmed by measuring the luciferase activity.

### MTT Assay

LNCaP and PC3 prostate cancer cells were transfected with miR-33b-3p mimic, inhibitor or either negative control for 24 h. After transfection, the cells (1×10^4^/well) were planted in 96-well plates and cultured for 72 or 96 h. The 3-(4, 5-dimethylthiazolyl-2)-2, 5-diphenyltetrazolium bromide (MTT) (5 mg/mL; Sigma, MO, USA) was added into each well, and dissolved in 150 μL DMSO (Sigma) after 4 h incubation. The absorbance was measured at a wavelength of 490 nm by using a microplate spectrophotometer (Bio-Rad, CA, USA).

### Western Blotting

Cells were lysed radioimmunoprecipitation assay (RIPA) buffer (10 mM Tris-HCl, 150 mM NaCl, 0.1% SDS, 0.8% Triton X-100) containing 1×protease inhibitor cocktail (Roche, Mannheim, Germany) (Roche, Mannheim, Germany). Western blotting was performed as described previously (24). The primary antibodies were mouse anti-GAPDH (KC-5G4) (Kangchen, Shanghai, China), β-actin (Santa Cruz, CA, USA) and DOCK4 (Proteintech, Wuhan, China). The secondary antibodies were from Cell Signaling Technology (MA, USA).

### Wound Healing Assay

Cells (3 × 10^5^/well) were planted in 6-well plate. For starvation, cells were cultured in the serum-free medium for 24 h. The scratch was made by using the 200 μL plastic pipette tip. The scratched cells were washed with 1×PBS to remove debris. The scratches were photographed after 24 h and 48 h incubation.

### Transwell Assay

Cell migration and invasion assays were performed using Transwell chambers with polycarbonate filter (8 μm pore) (Corning Costar, Cambridge, MA, USA). Cells were trypsinized and resuspended in serum-free medium. Cell suspensions (4 × 10^4^ cells in 150 μL) were seeded in the upper chamber, and 500 μL medium containing 20% FBS was added to the lower chamber. Cells were incubated at 37°C for 48 h. Cells remaining on the surface of the upper chamber were removed by a cotton swab while cells migrated into the lower chamber were fixed with methanol and stained with 0.5% crystal violet (Sinopharm Chemical Reagent, Shanghai, China).

For cell invasion assay, Transwell filters were pre-coated with 50 μL Matrigel (BD Biosciences, Franklin Lakes, NY, USA) at working concentration (0.3 mg/mL) in serum-free medium. The assay was performed following the migration assay protocol. The migrated and invasive cells were counted under microscope and quantified by Image J software (NIH, Bethesda, MD, USA).

### Dual Luciferase Reporter Assay

Prostate cancer cells (1×10^5^ cells/well) were grown in 24-well plate. The pcDNA3.1-miR-33b (0.3 μg) was co-transfected with pMIR-WT-3’ UTR (0.3 μg) or pMIR-MUT-3’ UTR of *DOCK4* (0.3 μg) in the presence of pRLSV40 (0.03 μg) into cells for 48 h. Luciferase activities were assessed by Dual-Luciferase Reporter Assay System (Promega, Fitchburg, WI, USA). Renilla luciferase activity was normalized to the firefly luciferase.

### RNA-Seq Analysis

Total RNA was extracted by Trizol (Ambion, CA, USA). Library preparation and transcriptome sequencing were performed by Novogene (Beijing, China). The library preparations were sequenced on an Illumina Novaseq platform and 150 bp paired-end reads were generated. The raw data of RNA-seq was deposited to the Gene Expression Omnibus (GEO) (GEO accession number: GSE183428).

Differential expression analysis of two groups (three biological replicates per group) was performed using the DESeq2 R package (1.20.0). The resulting P-value was adjusted using the Benjamini and Hochberg’s approach for controlling the false discovery rate. Genes with an adjusted *P*< 0.05 found by DESeq2 were assigned as differentially expressed.

Gene Ontology (GO) enrichment analysis of differentially expressed genes was implemented by the clusterProfiler R package, in which gene length bias was corrected. GO terms with corrected P< 0.05 were considered significantly enriched by differential expressed genes.

### *In Vivo* Study

All studies in animals were performed according to protocol approved by the Animal Care and Use Committee of Harbin Institute of Technology. BALB/c nude mice aged at 4 weeks were obtained from Beijing Vital River Laboratory Animal Technology Co., Ltd. and housed in a specific pathogen-free environment. The mice were inoculated with PC-3M-1E8-luciferase cells (1.5 × 10^6^) through tail vein, and luminescent signal was monitored every week. After 7 weeks of inoculation, the mice were sacrificed. The removed lung, liver, lymph nodes, and tumor tissues were fixed with 4% paraformaldehyde.

For the bortezomib treatment experiment, miR-33b-3p-overexpressing and control PC-3M-1E8-luciferase cells were injected into the mice through tail vein. After 30-days inoculation, each group was administrated with bortezomib (LC Labtoratories, MA, USA) (n=5) at a dose of 0.5 mg/kg body weight in 100 uL or equal volume of PBS (n=4), respectively. The drug was administered by intraperitoneal injection for four weeks, twice a week. Then, the mice were sacrificed except for 2 mice from bortezomib treated control group which were died after two weeks of treatment. The removed lungs were fixed with 4% paraformaldehyde. The samples from both experiments were embedded in paraffin and cut at 10 μM. Sections were deparaffinized and stained with hematoxylin and eosin (H&E), then mounted for microscope observation.

### Overall Survival Analysis

The overall survival curves of patients from TCGA was plotted by Kaplan-Meier method and the log-rank test. Patients were divided into high and low groups of miR-33b-3p expression according to the median value.

### Statistical Analysis

Microsoft Excel software was used for statistical analysis. Results for continuous variables are presented as means ± SD unless stated otherwise. For two-group comparison, two-tailed Student’s *t*-test was used.

## Results

### Downregulation of miR-33b-3p in Prostate Cancer Cells

**Figure 1:**
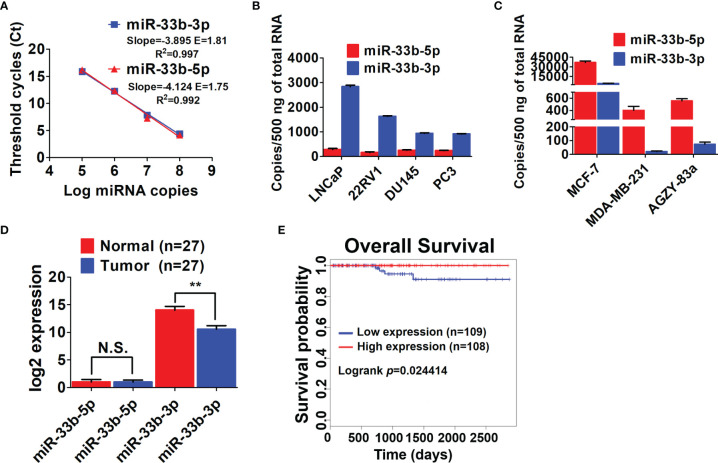
miR-33b-3p was downregulated in prostate cancer cells. **(A)** Ct values *versus* log miRNA copies were plotted to calculate the slope. The corresponding RT-PCR primers efficiencies were calculated according to the equation: E=10^[-1/slope]^. RT-qPCR analysis of both arms of miR-33b in prostate **(B)**, breast and lung cancer cell lines **(C)** as well as tumor and adjacent normal tissues of prostate adenocarcinoma patients (n=27) **(D)**. **(E)** Kaplan-Meier curve of overall survival of prostate adenocarcinoma patients analyzed by using TCGA. Blue and red curve represents patients with low or high expression of miR-33b-3p, respectively. Data **(A–C)** are representative of at least three independent experiments. N.S., not significant, **P < 0.01 by two-tailed Student’s *t-*test.

Our previous array analysis showed that the 3p arm of miR-33b was downregulated in response to proteasome inhibition by both the authentic inhibitor bortezomib and celastrol with proteasome inhibitory effect in prostate cancer (14, 25). As both arms of miRNAs can be processed into mature form depending on different cell context (17, 18), we determined the expression levels of the two arms of miR-33b in prostate cancer cell lines. Firstly, the efficiency of primers for both arms of miR-33b was evaluated, showing that the primers for miR-33b-3p and miR-33b-5p had equal amplification efficiency (**Figure 1A**). Then the copy number of per sample (500 ng reverse transcribed RNA) was calculated based on the Ct values by using the calibration curve of each arm of miR-33b. The results showed that miR-33b-3p was the major mature form in prostate cancer cell lines, while miR-33b-5p was almost undetected (**Figure 1B**). On the contrary, miR-33b-5p was the major form in breast and lung cancer cell lines (**Figure 1C**). Analysis of clinical samples indicated that both arms of miR-33b can be processed to mature forms; however, miR-33b-3p was the major isoform in normal prostate tissues as well as tumor samples (**Figure 1D**). In addition, miR-33b-3p level was downregulated in tumor tissues compared with the normal counterpart while no significant difference was observed in terms of miR-33b-5p level (**Figure 1D**). The overall survival analysis using the Kaplan-Meier curves demonstrated that prostate cancer patients with high level of miR-33b-3p exhibited significantly better survival (logrank *p*=0.024414) (**Figure 1E**). These results suggest that miR-33b-3p might act as tumor suppressor in prostate cancer.

### miR-33b-3p Inhibits Prostate Cancer Cells Proliferation

**Figure 2:**
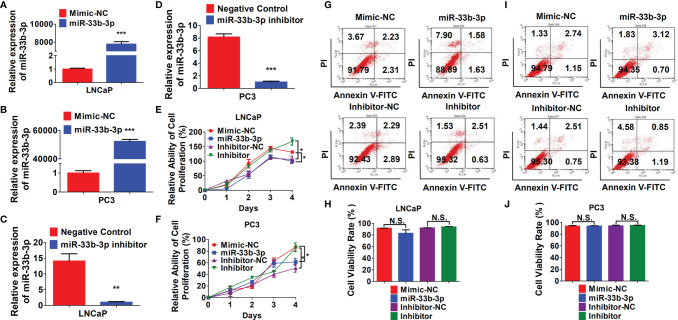
miR-33b-3p inhibits prostate cancer cells proliferation. **(A–F)** LNCaP and PC3 cells were transfected with miR-33b-3p mimics (miR-33b-3p), negative control of mimic (Mimic-NC), miR-33b-3p inhibitor (Inhibitor) or negative control of inhibitor (Inhibitor-NC) for 48 h, followed by RT-qPCR analysis **(A–D)**, MTT assay **(E**, **F)** and flow cytometry **(G–J)**. Data are representative of three independent experiments. Data are presented as mean ± SD. *P < 0.05, **P < 0.01, ***P < 0.001 by two-tailed Student’s t test. N.S., not significant.

To investigate the tumor suppressive role of miR-33b-3p in prostate cancer cells, miR-33b-3p mimic or inhibitor were transfected into the prostate cancer cells, and the efficacy of transfection was determined by RT-qPCR (**Figures 2A–D**). MTT assays showed that compared with the group transfected with the mimic control, transfection with miR-33b-3p mimic greatly reduced cell numbers in androgen receptor (AR) positive LNCaP and AR negative PC3 cells (**Figures 2E, F** **)**. Conversely, transfection with miR-33b-3p inhibitor led to enhanced cell proliferation in LNCaP and PC3 cells (**Figures 2E, F** **)**. Cell viability was not affected by altered miR-33b-3p expression levels, no matter overexpression or inhibition (**Figures 2G–J**).

### miR-33b-3p Inhibits Prostate Cancer Cells Migration and Invasion

**Figure 3:**
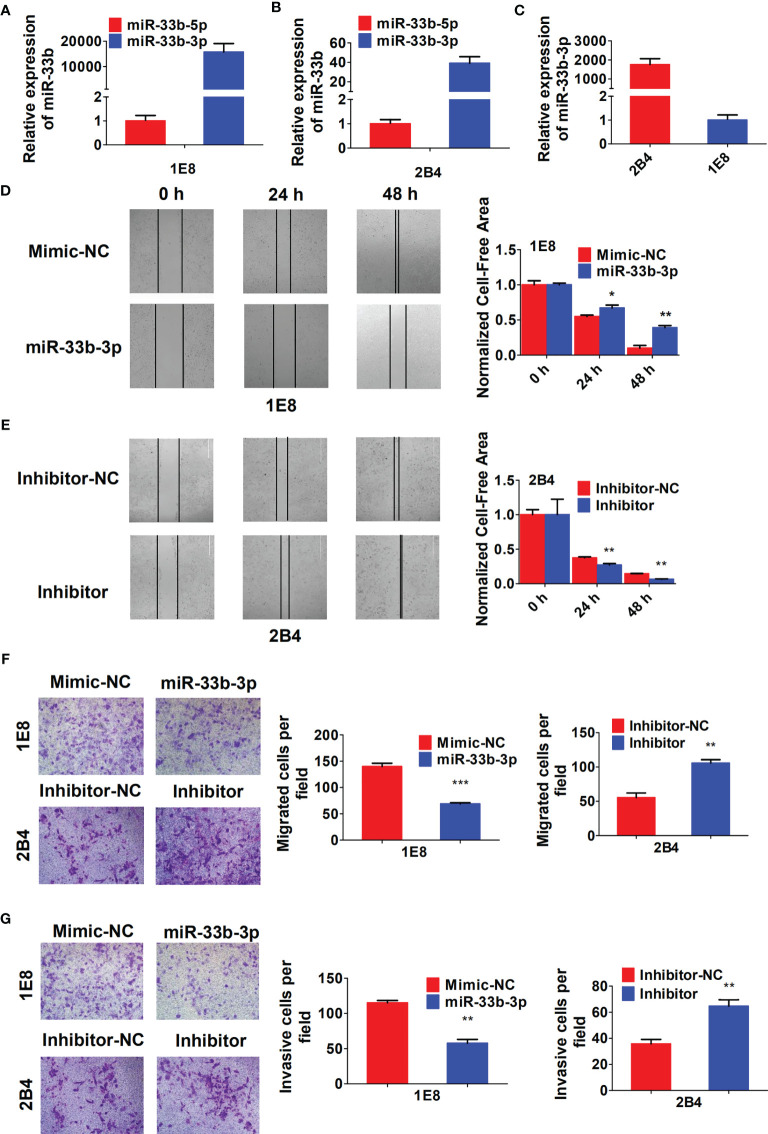
miR-33b-3p inhibits prostate cancer cells migration and invasion *in vitro*. **(A–C)** RT-qPCR analysis in PC-3M-1E8 and PC-3M-2B4 cells with highly or lowly metastatic ability, respectively. PC-3M-1E8 or PC-3M-2B4 cells were transfected with miR-33b-3p mimic or inhibitor for 48 h, followed by wound healing **(D, E)** and transwell assay to examine migration **(F)** and invasion **(G)**. Data are representative of at least three independent experiments. *P < 0.05, **P < 0.01, ***P < 0.001 by two-tailed Student’s *t* test.

To investigate the role of miR-33b-3p in migration and invasion of prostate cancer cells, the PC-3M-1E8 and PC-3M-2B4 cell lines that are derived from PC-3M prostate cancer cells but with different metastatic potentials were used as cell models (26). We confirmed that miR-33b-3p was still the major mature form in both PC-3M-1E8 and PC-3M-2B4 cells (**Figures 3A, B** **)**. In addition, miR-33b-3p level was found much lower in highly metastatic PC-3M-1E8 than that in the lowly metastatic PC-3M-2B4 cells (**Figure 3C**). Wound healing assay showed that PC-3M-1E8 cells with miR-33b-3p mimic transfection had slower recovery rates than control cells (**Figure 3D**). On the contrary, transfection with miR-33b-3p inhibitor in PC-3M-2B4 cells led to faster wound closure than that control group (**Figure 3E**). Transwell assays showed that overexpression of miR-33b-3p inhibited the migrative and invasive ability of highly metastatic PC-3M-1E8 cells. Conversely, transfection of miR-33b-3p inhibitor accelerated PC-3M-2B4 cells invasion and migration (**Figures 3F, G** **)**. Together, these results demonstrate that miR-33b-3p can inhibit the invasion of prostate cancer cells *in vitro*.

### miR-33b-3p Inhibits Prostate Cancer Metastasis *In Vivo*

**Figure 4:**
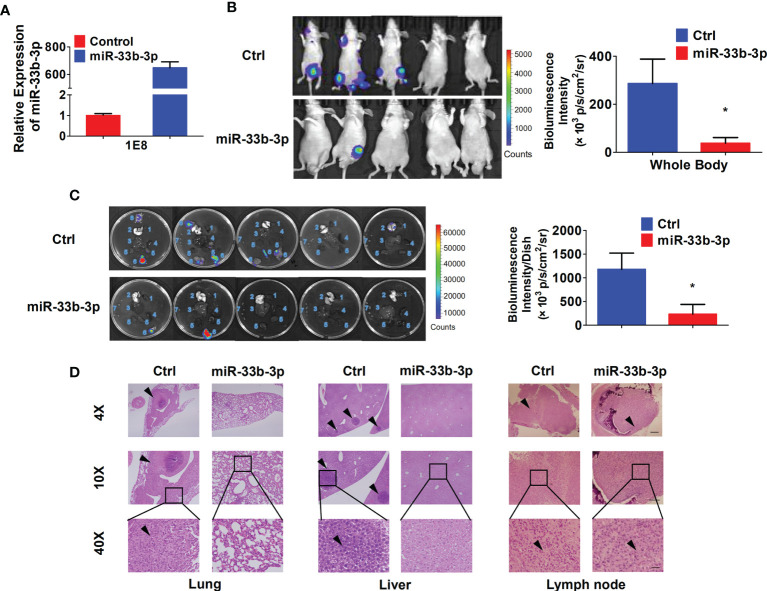
miR-33b-3p inhibits prostate cancer metastasis *in vivo*. **(A)** RT-qPCR analysis in PC-3M-1E8-luciferase cells. **(B, C)** miR-33b-3p overexpression or control PC-3M-1E8-luciferase cells (1.5 × 10^6^ per inoculation) were injected into BALB/c nude mice through tail vein (n = 5). Mice were sacrificed after 7 weeks, and bioluminescent intensity (photons/sec/cm2/steradian) of whole body **(B)** and removed organs **(C)** were detected and analyzed *via* D-luciferin. 1 Heart, 2 Lung, 3 Liver, 4 Spleen, 5 Kidneys, 6 Lymph nodes with luminescence signal, 7 Lymph nodes without luminescence signal, 8 Cyst. **(D)** H&E staining. Arrowheads indicate cancer metastasis foci. The scale bar represents 4×, 10 mm; 10×, 5 mm; and 40×, 1 mm. *P < 0.05.

To investigate whether miR-33b-3p could suppress metastasis *in vivo*, the *luciferase* reporter gene was introduced into PC-3M-1E8 cells to trace tumor metastasis. The PC-3M-1E8 expressing luciferase cells were transfected with pLKO.1-miR-33b-3p or pLKO.1 vector. Overexpression of miR-33b-3p was confirmed (**Figure 4A**), then the cells were injected into BALB/c nude mice through tail vein, and metastasis of cancer cells was monitored *via* D-luciferin. Luminescence imaging showed that overexpression of miR-33b-3p led to significant decrease in luminescent intensity in the whole body as well as the removed organs (**Figures 4B, C** **)**. Dissection of mice organs further revealed that lung metastasis was detected in the control group but not miR-33b-3p group (**Figure 4C**). In addition, more lymph nodes metastasis was detected in the control group (7 luminescent lymph nodes in 3 mice) compared to miR-33b-3p group (2 luminescent lymph nodes in 2 mice) (**Figure 4C**). HE staining confirmed that lung metastasis was only observed in the control group (**Figure 4D**). Moreover, PC-3M-1E8 cells extensively metastasized to the lymph nodes and liver in the control group, whereas it was significantly suppressed in miR-33b-3p group (**Figure 4D**). These results demonstrate that miR-33b-3p exerts its anti-metastasis function *in vivo*. We further analyzed expression of miR-33b-3p in prostate cancer patients with different TNM stages using TCGA datasets (http://www.cbioportal.org/). The expression of miR-33b-3p had no difference between different T or M stages in prostate cancer patients (**Supplementary Figures 3A, B** **)**.

### miR-33b-3p Targets DOCK4 to Regulate Cell Motility in Prostate Cancer

**Figure 5:**
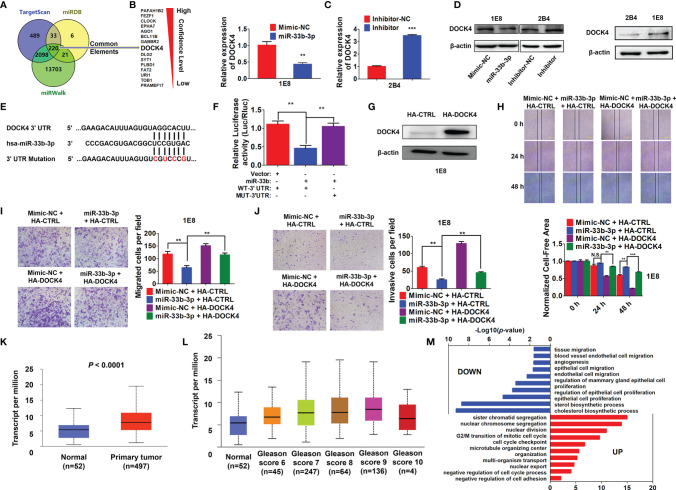
miR-33b-3p targets *DOCK4* to regulate cell motility in prostate cancer.** (A)** The top 15 targets of miR-33b-3p were predicted by miRPathDB, TargetScan and miRWalk. **(B, C)** RT-qPCR and Western blotting analysis of *DOCK4* in PC-3M-1E8 and PC-3M-2B4 cells post transfection with indicated oligoes. **(D)** Comparison of DOCK4 level between PC-3M-1E8 and PC-3M-2B4 cells. **(E, F)** Luciferase reporter assay. Luciferase reporter constructs containing wild-type (WT-3’UTR) or mutation (MUT-3’UTR) in the 3’ UTR of *DOCK4* were generated **(E)** and co-transfected with pcDNA3.1-miR-33b (miR-33b) or pcDNA3.1 empty vector (Vector) for 24 h. Luciferase activity was determined **(F)**. **(G)** Western blotting analysis of DOCK4 protein level in DOCK4 overexpression (HA-DOCK4) and control (HA-CTRL) PC-3M-1E8 cells. **(H–J)** DOCK4 overexpression and control PC-3M-1E8 cells were transfected with mimic negative control (Mimic-NC) or miR-33b-3p mimics (miR-33b-3p) for 48 h, followed by wound healing **(H)** and Transwell assays **(I, J)**. **(K)** The expression of *DOCK4* in TCGA. **(L)** The expression of *DOCK4* based on patient’s gleason score analyzed by using TCGA. **(M)** Gene Ontology (GO) functional enrichment (Biological Process, BP) analysis of differentially expressed genes between the control group and miR-33b-3p overexpression group. The value -log10(*p*-value) > 1.5 was used as the threshold to select GO terms. Data **(B, F–J)** are representative of at least three independent experiments. N.S., not significant, **P < 0.01, ***P < 0.001 by two-tailed Student’s *t*-test. The statistical significance of Normal-*vs*-Primary is 8.68299876444212E-11 **(K)**. The statistical significance of Normal-*vs*-Gleason score 6 is 1.435520E-03; Normal-*vs*-Gleason score 7 is 3.24329999523698E-08; Normal-*vs*-Gleason score 8 is 7.61050000019914E-07; Normal-*vs*-Gleason score 9 is 1.48140388844809E-11; Gleason score 6-*vs*-Gleason score 8 is 1.363590E-02; Gleason score 6-*vs*-Gleason score 9 is 2.406600E-04; Gleason score 7-*vs*-Gleason score 9 is 3.778400E-03 **(L)**.

To further elucidate the underlying molecular mechanism, three online prediction softwares (TargetScan, miRWalk and miRPathDB) were used to predict miR-33b-3p targets. Total 220 target genes were predicted through integration of the three programmes (**Figure 5A**). The top 15 potential targets of miR-33b-3p were selected based on the confidence level (**Figure 5A**). Among them, the expression level of *DOCK4* was the most different between normal and prostate cancer tissues (**Table 2**). Overexpression of miR-33b-3p led to deceased expression of *DOCK4* in both mRNA and protein levels in highly metastatic PC-3M-1E8 cells, while inhibition of miR-33b-3p could enhance the expression of *DOCK4* in lowly metastatic PC-3M-2B4 cells (**Figures 5B, C** **)**. Moreover, PC-3M-1E8 cells with low level of miR-33b-3p had high protein level of DOCK4 in relative to PC-3M-2B4 cells (**Figure 5D**), indicating that endogenous miR-33b-3p level had an influence on DOCK4 expression.

**Table 2:** Expression of potential target genes of miR-33b-3p in prostate cancer.

Potential genes	TCGA samples		Normal-*vs*-Primary tumor
	Normal (n = 52)	Primary tumor (n = 497)	
	Median	Median	Statistical significance
PAFAH1B2	35.13	23.57	1.46E-06
FEZF1	0.00	0.00	2.52E-07
CLOCK	5.12	3.26	5.60E-03
EPHA7	5.43	3.17	9.36E-04
AGO1	12.74	12.24	6.82E-02
BCL11B	0.46	0.32	7.58E-02
GABBR2	0.05	0.03	5.05E-01
**DOCK4**	**5.39**	**7.73**	**8.68E-11**
DLG2	3.12	2.08	5.60E-03
SYT1	5.80	1.97	1.81E-02
PLBD1	15.00	4.51	9.39E-11
FAT2	4.42	0.74	1.01E-07
URI1	68.03	69.63	4.62E-04
TOB1	144.84	123.62	8.26E-03
PRAMEF17	0.00	0.00	N/A

The data generated by TCGA Research Network (http://cancergenome.nih.gov/) has been used for UALCAN analysis (http://ualcan.path.uab.edu). Significance of difference estimated by Student’s t-test.

N/A, not applicable. The most upregulated gene DOCK4 in prostatae tumor tissues was highlighted with bold.

To determine whether miR-33b-3p directly regulated *DOCK4* expression, luciferase reporters carrying the wild type or mutation of miR-33b-3p binding site in the 3’ UTR of *DOCK4* were constructed (**Figure 5E**). Dual luciferase assays showed that miR-33b-3p suppressed the luciferase activity of the reporter containing the wild type 3’ UTR of *DOCK4* in PC-3M-1E8 cells, and this effect was abrogated with the mutated reporter (**Figure 5F**). These results indicate that *DOCK4* is a target of miR-33b-3p in prostate cancer cell lines. Whether miR-33b-3p exerted the motility-suppressing roles by targeting *DOCK4* in prostate cancer was determined. *DOCK4* was overexpressed in PC-3M-1E8 cells (**Figure 5G**). Wound healing assay showed that the inhibition of wound closure caused by miR-33b-3p overexpression was restored back to control level when *DOCK4* was overexpressed (**Figure 5H**). Consistently, *DOCK4* overexpression significantly antagonized the inhibitory effects of miR-33b-3p on the migration and the invasion in PC-3M-1E8 cells (**Figures 5I, J** **)**. Furthermore, overexpression of DOCK4 was observed in primary prostate tumors compared with normal tissues in TCGA dataset (**Figure 5K**). In addition, the expression of DOCK4 was upregulated along with the increased patient’s gleason score (gleason score 6-9) (**Figure 5L**). These results indicate that the motility-suppressing effects of miR-33b-3p on prostate cancer cells were associated with downregulation of *DOCK4*. Analysis of *DOCK4* expression in prostate cancer patients using TCGA and UALCAN datasets (http://ualcan.path.uab.edu/index.html) showed that the level of *DOCK4* had no difference between different T or N stages (**Supplementary Figures 4A, B** **)**. Analysis of disease-free survival using the Gene Expression Profiling Integrative Analysis (GEPIA; http://gepia.cancer-pku.cn) showed that high expression of *DOCK4* in prostate cancer tissues was associated with a shorter lifespan of patients (p = 0.032) (**Supplementary Figure 4C**), indicating that high level of *DOCK4* correlates with poor prognosis of prostate cancer patients.

To investigate the global changes of biological processes influenced by miR-33b-3p, we overexpressed miR-33b-3p in PC-3M-1E8 cells and performed RNA-Seq. As compared with control, enforced expression miR-33b-3p in prostate cancer cells led to high enrichment of several terms that downregulate cholesterol biosynthesis, cell proliferation and cell motility as well as angiogenesis (**Figure 5M**). These results indicate that miR-33b-3p regulates the extensive biological processes that are associated with prostate cancer cells homing and growth in the metastatic sites.

### Overexpression of miR-33b-3p Enhances Bortezomib Effects

**Figure 6:**
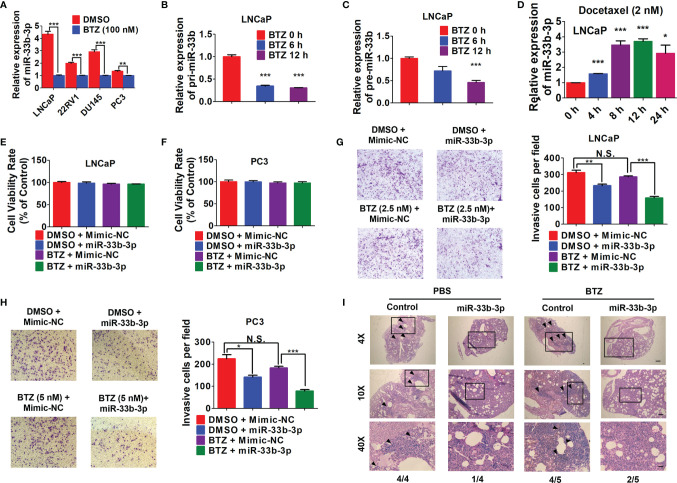
Overexpression of miR-33b-3p enhances bortezomib effects. **(A)** RT-qPCR analysis of miR-33b-3p expression in prostate cancer cells post treatment with bortezomib (BTZ) (100 nM) for 24 h. **(B, C)** RT-qPCR analysis the expression of pri- miR-33b and pre-miR-33b in LNCaP cells treated with BTZ (100 nM) for up to 12 h. **(D)** Relative expression level of miR-33b-3p in LNCaP cells treated with Docetaxel (2 nM) for up to 24 h. **(E–H)** miR-33b-3p overexpression and control cells were treated with or without BTZ (2.5 nM in LNCaP, 5 nM in PC3 cells) for 48 h, followed by flow cytometry **(E, F)** and Transwell assays **(G, H)**. **(I)** H&E staining in lung tissues. miR-33b-3p overexpression (miR-33b-3p) and control PC-3M-1E8-luciferase cells were injected into the mice through tail vein. After 30-days inoculation, each group was administrated with BTZ (n=5) at a dose of 0.5 mg/kg body weight in 100 uL or equal volume of PBS (n=4), respectively. Removed lungs from each group were stained by H&E. The numbers of mice developed lung metastasis were indicated. Arrowheads indicate cancer metastasis foci. The scale bar represents 4×, 10 mm; 10×, 5 mm; and 40×, 1 mm. Data **(A–H)** are representative of three independent experiments. N.S., not significant, *P < 0.05, **P < 0.01, ***P < 0.001 by two-tailed Student’s *t-*test.

Given that bortezomib demonstrated efficacy against multiple myeloma but not solid cancers, we speculated that bortezomib-induced miR-33b-3p inhibition might contribute to its inefficacy in prostate cancer. We confirmed that bortezomib inhibited miR-33b-3p expression in prostate cancer cell lines (**Figure 6A**). qRT-PCR indicated that the expression of pri-miR-33b and pre-miR-33b was also downregulated by bortezomib (**Figures 6B, C** **)**. Docetaxel is the clinically used chemotherapeutics of mCRPC. As an effective agent, treatment with docetaxel increased the expression of miR-33b-3p in LNCaP cells (**Figure 6D**). These results suggest that the downregulation of miR-33b-3p might be associated with bortezomib’s inefficacy in prostate cancer cells. Next, we determined whether bortezomib-induced miR-33b-3p inhibition might contribute to its inefficacy in prostate cancer. Based on MTT assay (**Supplementary Figure 1A**), non-cytotoxic dose of bortezomib was used to treat LNCaP and PC3 cells for transwell assays. Cell death assay confirmed that bortezomib did not affect cell viability in miR-33b-3p or control treated cells (**Figures 6E, F** **)**. Transwell assays indicated that overexpression of miR-33b-3p (**Supplementary Figures 2A, B** **)** promoted the inhibitory effect of bortezomib on motility of prostate cancer cells (**Figures 6G, H** **)**.

To further determine whether overexpression of miR-33b-3p could influence the effect of bortezomib on the metastasis of prostate cancer *in vivo*, miR-33b-3p-overexpressing and control PC-3M-1E8-luciferase cells were injected into the mice through tail vein. After 30-days inoculation, each group was administrated with bortezomib or equal volume of PBS, respectively. After four weeks, the mice were sacrificed. H&E staining confirmed the development of lung metastasis in four groups (**Figure 6I**). The summarize of H&E results showed that the control group receiving PBS all developed lung metastasis (4/4), while control group receiving bortezomib developed lung metastasis in 4/5. Again, we observed that miR-33b-3p inhibited metastasis (1/4 lung metastasis in miR-33b-3p group receiving PBS) and over-expression of miR-33b-3p could enhanced bortezomib effects on suppression of lung metastasis (2/5). This result indicates that bortezomib had limited effect on metastasis suppression in prostate cancer which can be enhanced by miR-33b-3p.

## Discussion

During miRNA biogenesis, the 5p strand of miRNA duplex is usually loaded into RISC and selected as dominant mature miRNA (27). Here, we found that miR-33b-3p was the dominant mature form in prostate cancer. miR-33b-3p were expressed over miR-33b-5p in prostate cancer cell lines and tumor tissues. It is unclear how prostate cancer cells select miR-33b-3p as the dominant mature form in the current study. Recent study indicates that high level of the terminal uridylyl transferases TUT4 and TUT7 leads to selection of the 3p strand instead of the 5p strand of miR-324 (28). In addition, target mRNA of miRNA also determines the arm selection of miRNA (18). Given that miR-33b-5p could be detected as a major mature form in other type of cancer tissues (29, 30), we deduced that miR-33b-3p selection in prostate cancer might depend on the prostate cell type.

Recently, miRNAs were implicated in cancer metastasis and emerged as “metastamir” or metastasis suppressor (31–33). miR-33b-3p is recognized as an oncogenic or tumor suppressive molecule depending on different cell context. It targets the well-known tumor suppressor CDKN1A (p21) and contributes to cisplatin-resistance in lung cancer (34). Overexpression of miR-33b-3p enhanced cell proliferation and colony formation in gastric cancer (35), indicating the oncomir function of miR-33b-3p. On the other hand, downregulation of miR-33b-3p was found associated with aggressive phenotype of bladder cancer (22). In addition, decreased level of circulating miR-33b-3p was detected in patients with upper tract urothelial carcinoma in comparison with normal control group (36), supporting the tumor suppressive role of miR-33b-3p. In line with these observations, our results demonstrate that miR-33b-3p is a metastasis suppressor in prostate cancer. Low expression level of miR-33b-3p was detected in patients with prostate cancer and associated with their shortened survival period. Overexpression of miR-33b-3p suppressed whereas inhibition of miR-33b-3p promoted the migration and invasion abilities of cancer cells. Furthermore, *in vivo* study confirmed the anti-metastasis function of miR-33b-3p, showing that miR-33b-3p decreased the metastasis of prostate cancer cells to lymph nodes, lung, as well as liver. In this study, miR-33b-3p also inhibit the proliferation of prostate cancer cells. Our RNA-Seq data indicate that miR-33b-3p suppresses biological process such as angiogenesis and endothelial cell migration, suggesting that miR-33b-3p might regulate extensive pathways involved in prostate cancer cells homing and growth in the metastatic sites. We cannot eliminate the influence of these biological processes on metastasis, which is the limitation of our current study.

The current study demonstrates that miR-33b-3p inhibits metastasis by targeting *DOCK4* in prostate cancer. Overexpression of *DOCK4* abolished the inhibition of miR-33b-3p on migration and invasion in prostate cancer cells. DOCK4 is a member of the DOCK180 family of guanine nucleotide exchange factor. It was first identified as a Rap GTPase activator that can enhance the formation of adherent junctions between cells, mutations of which was found to confer the loss of cell junction and an invasive phenotype in cancer (37). However, subsequent studies indicate that DOCK4 might promote cell invasion rather than cell-cell adhesion. Overexpression of DOCK4 in placental cytotrophoblasts increased the invasiveness of these cells (38). Upregulation of DOCK4 was found associated with metastases of breast and lung cancer (39, 40). In this study, overexpression of wild type of *DOCK4* promoted the migration and invasion of prostate cancer cells, supporting its function to mediate metastasis in prostate cancer.

The proteasome is a validated target for cancer therapy. Proteasome inhibition represents a successive strategy for the treatment of multiple myeloma. However, proteasome inhibitors failed to benefit to patients with solid tumors for some reasons (41). The current study suggests that downregulation of miR-33b-3p is one of the reasons resulting in bortezomib limited efficacy in prostate cancer. Under non-cytotoxic doses, bortezomib had no effect on inhibition of cell invasion, while overexpression of miR-33b-3p could improve bortezomib suppression on migration and invasion. Therefore, the current study suggests that overcoming miR-33b-3p downregulation might increase bortezomib efficacy against prostate cancer.

In summary, our results demonstrate the anti-tumor function of miR-33b-3p in prostate cancer. It suppresses the invasion and migration of prostate cancer cells by targeting *DOCK4*. Proteasome inhibitor bortezomib leads to insufficient inhibition on cell invasion due to miR-33b-3p downregulation, which can be overcome by overexpression of miR-33b-3p in prostate cancer.

## Data Availability Statement

The datasets presented in this study can be found in online repositories. The names of the repository/repositories and accession number(s) can be found below: NCBI GEO repository, GSE183428.

## Ethics Statement

The animal study was reviewed and approved by Animal Care and Use Committee, Harbin Institute of Technology

## Author Contributions

HuY and YM conceived the project, analyzed the data, and wrote the paper. YM and KL designed and performed the experiments. ML and SS contributed to the *in vivo* experiments. ZZ, ML, HuY, HW, XH and XL helped other experiments. All authors contributed to the article and approved the submitted version.

## Funding

This work was supported by National Natural Science Foundation of China (81872439), Natural Science Foundation of Heilongjiang Province (LH2020H072) and Innovation Research Project of Harbin (2017RAQXJ182).

## Conflict of Interest

The authors declare that the research was conducted in the absence of any commercial or financial relationships that could be construed as a potential conflict of interest.

## Publisher’s Note

All claims expressed in this article are solely those of the authors and do not necessarily represent those of their affiliated organizations, or those of the publisher, the editors and the reviewers. Any product that may be evaluated in this article, or claim that may be made by its manufacturer, is not guaranteed or endorsed by the publisher.
