# Supplementary material for: miR-33b-3p Acts as a Tumor Suppressor by Targeting DOCK4 in Prostate Cancer
Source: Front Oncol. 2021 Nov 3;11:740452. doi: 10.3389/fonc.2021.740452 (PMC8595470; doi:10.3389/fonc.2021.740452)
Supplement: Supplementary file 1 [file DataSheet_1.pdf]

Table S1. The differentially expressed genes (|log2FoldChange| > 2) in 1E8-Mimics-NC and 1E8-miR-33b-3p cells.

Gene Symbol	mRNA Expression Raw Intensities		Log2 Fold change
	1E8-miR-33b-3p	1E8-mimic-NC	(1E8-miR-33b-3p / 1E8-mimic-NC)
CBSL	31.72498	0.98914	5.000288
FBLL1	4.682745	0.335926	3.716041
AC004466.3	4.346858	0.335926	3.608592
NPIPA2	4.320457	0.327267	3.601436
ZFX-AS1	4.30206	0.335926	3.596333
SFR1P1	4.025364	0.330937	3.496801
AC007277.1	4.01381	0.335926	3.493482
PPIAP51	3.680052	0.335926	3.368166
AC067931.2	5.32102	0.658204	3.009428
AC024558.1	8.972541	1.321396	2.76174
PLA1A	6.347985	0.99046	2.676135
AL035665.1	5.96872	0.99046	2.589791
NPIPB6	5.951743	0.994129	2.584451
AC100827.4	5.664913	0.994129	2.510895
CASC15	10.69413	1.9796	2.429081
AC009032.1	7.004657	1.330055	2.399931
ABHD14A-ACY1	6.682453	1.326385	2.332905
RAB28P5	6.664057	1.333725	2.326624
EPX	8.032332	1.652333	2.277778
ST7-OT4	8.980546	1.991929	2.175091
PGLYRP1	10.97483	2.668769	2.045761
AC091825.1	9.397314	2.317876	2.017077
TMEM161BP1	0.333758	4.328442	-3.59271
LINC00163	0.333758	3.985177	-3.47415
COCH	0.667516	6.019369	-3.17129
GRIK1	0.982877	7.624449	-2.93863
FP236383.1	1.000564	6.949959	-2.79673
LINC01144	1.01754	6.620341	-2.71713
LINC01933	1.656525	8.967121	-2.43019
TGIF2P1	1.661948	8.67449	-2.37919
AC009962.1	1.345876	6.945258	-2.37595
RPL7AP11	1.334322	6.608012	-2.30937
RPLP1P6	1.988863	9.310387	-2.22105
AC005759.1	1.66808	7.618429	-2.19179
AC139272.1	3.646099	16.3365	-2.15831
HAP1	2.680908	11.61461	-2.11845
AC055822.1	1.988863	8.271643	-2.05201
