# Supplementary material for: miR-33b-3p Acts as a Tumor Suppressor by Targeting DOCK4 in Prostate Cancer
Source: Front Oncol. 2021 Nov 3;11:740452. doi: 10.3389/fonc.2021.740452 (PMC8595470; doi:10.3389/fonc.2021.740452)
Supplement: Supplementary file 2 [file DataSheet_2.pdf]

miR-33b-3p Acts as a Tumor Suppressor by Targeting *DOCK4* in Prostate Cancer

Supplementary Figures

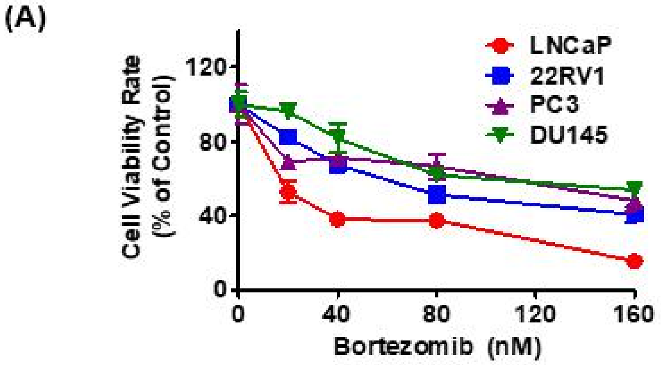

Supplementary Figure 1 | Cell viability post bortezomib treatment in prostate cancer cells. **(A)** LNCaP, 22RV1, PC3 and DU145 cells were treated with bortezomib up to 160 nM for 72 h, and cell viability was analyzed by MTT assay.

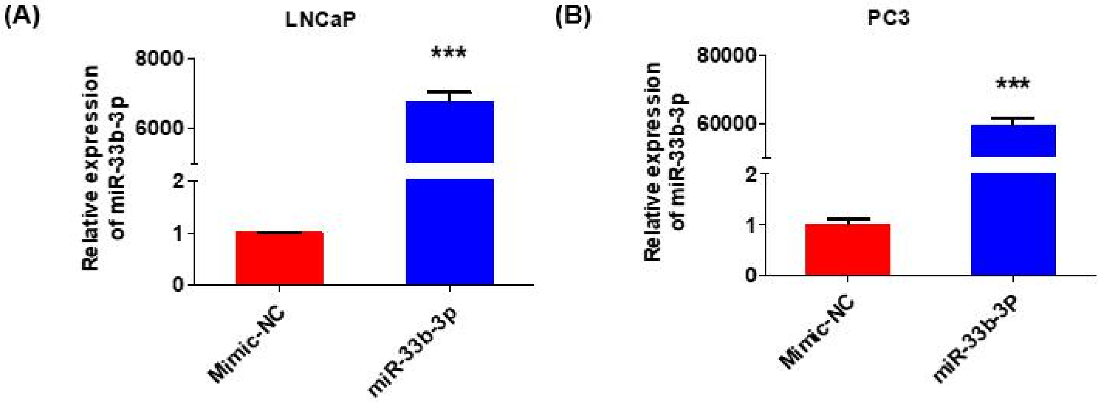

Supplementary Figure 2 | Overexpression of miR-33b-3p. RT-qPCR analysis of miR-33b-3p expression in LNCaP **(A)** and PC3 **(B)** cells transfected with miR-33b-3p

mimcs (miR-33b-3p) or negative control (mimic-NC) for 48 h. Data are presented as mean ± SD. ***$P < 0.001$ by two-tailed Student's *t*-test.

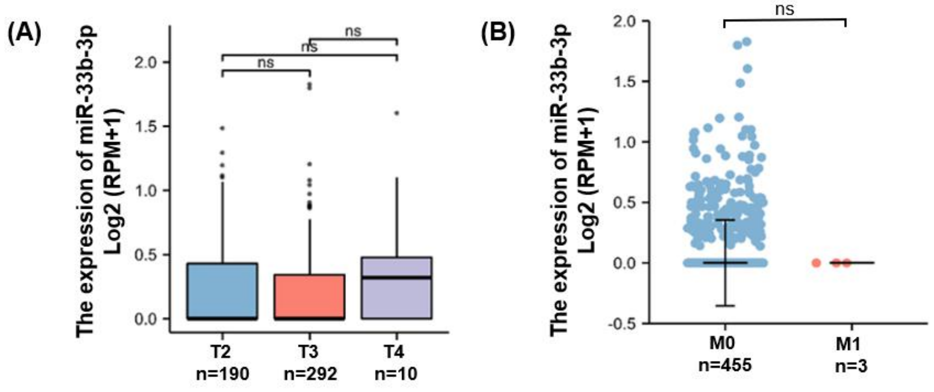

Supplementary Figure 3 | The expression of miR-33b-3p in prostate cancer patients with different TNM stages. The expression of miR-33b-3p in prostate cancer patients with different T stages **(A)** and M stage **(B)** in TCGA. ns, no significance.

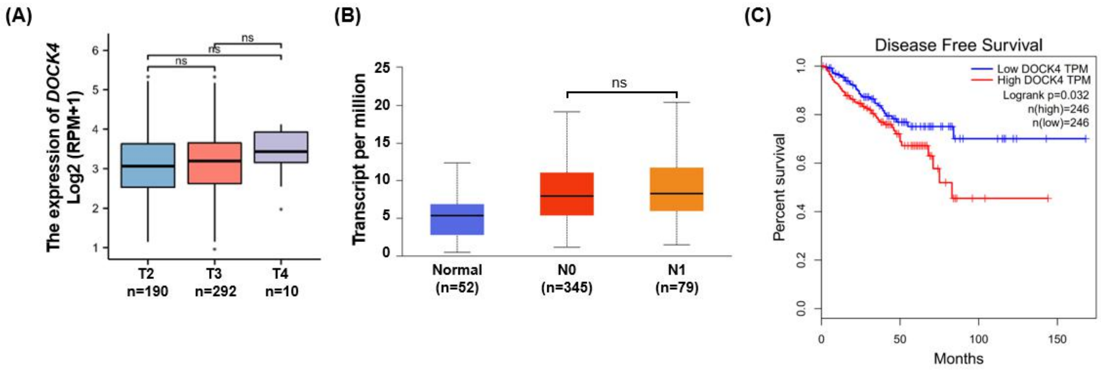

Supplementary Figure 4 | The expression of *DOCK4* and survival analysis in prostate cancer patients with different TNM stages. The expression of *DOCK4* in prostate cancer patients with different T stages in TCGA **(A)** and N stage in UALCAN datasets **(B)**. ns, no significance. **(C)** Kaplan-Meier curve of disease-free

survival of prostate cancer patients analyzed by using GEPIA. Blue curve represents patients with low expression of *DOCK4*, red curve represents patients with high expression of *DOCK4*.
